# Hernia, with Remarks

**Published:** 1869-03

**Authors:** C. C. F. Gay

**Affiliations:** Buffalo


ART. II.—*Hernia, with remarks.* By C. C. F. Gay, M. D., *Buffalo.*

1. *Strangulated Femoral Hernia.*—I was requested by Dr. Burwell to visit a patient for him, as he was called out of town and was uncertain at what hour he should return. In his note to me he advises an operation.

The patient was a married woman, aged forty-eight years, obese. I first saw her at 7½ P. M. The hernial protrusion was upon the right side, was caused by a fall from her bed, had existed forty-eight hours, and the taxis had been resorted to ineffectually. In the mean time an officious woman had administered a dose of castor oil and two or three doses of senna and salts. The patient was vomiting; bowels much distended; pulse 120 per minute and feeble.

At 9 o'clock P. M., assisted by Drs. Lothrop and Wetmore, the patient chloroformed, I cut down upon the sac, which did not appear to be invested with the usual coverings, divided the sac, and after considerable search found the stricture at Gimbernat's ligament, which was divided by the use of Sir Astley Cooper's hernia knife, the protruding bowel returned within the abdominal cavity, a wetted compress applied and morph. gr. ss administered.

At my visit next morning learned that soon after leaving my patient after the operation, that she had a copious evacuation of the bowels, and that the contents of the bowels had continued to run off during the night time. No abdominal tenderness or tympanitis; pulse 108. Morphia continued every six hours. At 8½ P. M. patient pulseless, and died at 12 o'clock—fifty hours after the operation.

2. *Strangulated Scrotal Hernia—direct—left side.*

W. T., aged fifty years, married, American, intemperate. This is an old hernia of twenty years' standing. The patient has been on a debauch for some days, and by his neglect of his hernial protrusion it has become strangulated. Dr. Strong, who kindly invited me to visit this patient along with him, had found taxis insufficient to restore the intestine. After making trial myself, and failing, and believing further efforts at reduction would do more harm than good, advised an operation. At 2 o'clock P. M. the patient was etherized and the operation made in the presence of several medical gentlemen.

In this case also the sac was divided, which discharged considerable fluid; the bowel was restored, but the opening through the external ring is now so large that the tendency to protrusion exists. Brought integuments together by sufficient number interrupted sutures, applied compress, suspensory bag for the testicles, and administered morph. gr. j, and ordered morphia gr. ss every three hours.

At our visit next morning learned that the patient had slept nearly seven hours during the night, but had vomited considerably, and could take no nourishment; pulse 100 per minute. Anticipating delirium tremens, whisky, morphia and beef essence were ordered. At 5 P. M. stomach rejects everything; pulse 124. Dr. S. directed tr. opii ℨ ij to be given by enema, and to be repeated every four hours. Stomach rejects everything, even champagne nauseates him. The epigastrium was blistered. Patient has had some days a very troublesome cough. There is no tympanitis now, two days after the operation; pulse 120; morphia sprinkled upon the vesicated surface.

Third day after the operation; much the same this morning. Last evening said his room was full of worms, was delirious in night time, could not keep him in bed. This morning is calmer, pulse more frequent. Continued treatment. Patient is able to take broth and stimulants; add quinine. Delirious again in evening, and died suddenly on the night of the fourth day after the operation.

Remarks.—Authors, I believe, all agree that the sooner an operation is made for strangulated intestine, after judicious trial of the taxis has been resorted to without success, the greater will be the chances of the patient to live, and the longer the operation is delayed the greater will be the chances of the patient to die; therefore, taxis should not be too often repeated nor too long applied. After a single well-directed effort at reduction of the protruding intestine, made by a single individual, without success, it were then better to advise and make an operation than to imperil the life of an individual by delay and unnecessary manipulation of the tender protruding part.

Had the operation in case of femoral protrusion herein reported, been made within twelve or twenty-four hours, I should be warranted in stating that the patient would have recovered. Recovery, too, might have taken place even though the operation was delayed more than forty-eight hours after strangulation, provided the intestines could afterwards have been at rest, and their peristaltic action had not been excited by hypercatharsis.

In the case of scrotal hernia, it was absolutely certain, and so stated at the time, that the patient would have delirium tremens whether the operation was made or not; such were the fears of the patient himself. Here, then, was a dilemma. If left without an operation he must certainly die from intestinal strangulation, and if operated upon death was almost as certain to ensue from delirium tremens. Yet with so small a chance in favor of the preservation of life, no one I suspect would hesitate to advise an operation.

In an article on hernia, published in the January number of the JOURNAL, the author of which is my ancient friend, Dr. Gibbs, I learn that he has had no occasion to use the knife for twenty years. He simply dilates the stricture with his finger thrust through the canal and within the ring, and eats his breakfast and the patient is cured. This is certainly a simple method for the cure of strangulated hernia, and should it ever supercede the necessity for the use of the knife, or the labor in teaching and studying the anatomy of the parts involved, there would be abundant reason for mutual congratulations.

The remark is often made that members of our profession much less willingly report their unsuccessful than their successful cases, or in other words that the physicians and surgeons report at once and promptly their cases that terminate favorably, while all operations attended by fatal result are not reported at all; now this should not be so. Report of cases are for instruction. Much more instruction may be derived from report of a fatal than from a successful case, and the profession is entitled to the details of any case from which instruction may be drawn.

With these brief remarks I close the report of two unsuccessful cases of operation for hernia with the promise that I shall be just as prompt and willing to report my successful cases.

